# FBXO16-mediated hnRNPL ubiquitination and degradation plays a tumor suppressor role in ovarian cancer

**DOI:** 10.1038/s41419-021-04040-9

**Published:** 2021-07-31

**Authors:** Mei Ji, Zhao Zhao, Yue Li, Penglin Xu, Jia Shi, Zhe Li, Kaige Wang, Xiaotian Huang, Jing Ji, Wei Liu, Bin Liu

**Affiliations:** 1 Department of Gynecology, The First Affiliated Hospital of Zhengzhou University, Zhengzhou, 450052 China; 2 Jiangsu Key Laboratory of Marine Pharmaceutical Compound Screening, College of Pharmacy, Jiangsu Ocean University, Lianyungang, 222005 China

**Keywords:** Ovarian cancer, Oncogenes

## Abstract

Heterogeneous nuclear ribonucleoprotein L (hnRNPL) is a type of RNA binding protein that highly expressed in a variety of tumors and plays a vital role in tumor progression. However, its post-translational regulation through ubiquitin-mediated proteolysis and the cellular mechanism responsible for its proteasomal degradation remains unclear. F-box proteins (FBPs) function as the substrate recognition subunits of SCF ubiquitin ligase complexes and directly bind to substrates. The aberrant expression or mutation of FBPs will lead to the accumulation of its substrate proteins that often involved in tumorigenesis. Here we discover FBXO16, an E3 ubiquitin ligase, to be a tumor suppressor in ovarian cancer, and patients with the relatively high expression level of FBXO16 have a better prognosis. Silencing or depleting FBXO16 significantly enhanced ovarian cancer cell proliferation, clonogenic survival, and cell invasion by activating multiple oncogenic pathways. This function requires the F-box domain of FBXO16, through which FBXO16 assembles a canonical SCF ubiquitin ligase complex that constitutively targets hnRNPL for degradation. Depletion of hnRNPL is sufficient to inactive multiple oncogenic signaling regulated by FBXO16 and prevent the malignant behavior of ovarian cancer cells caused by FBXO16 deficiency. FBXO16 interacted with the RRM3 domain of hnRNPL via its C-terminal region to trigger the proteasomal degradation of hnRNPL. Failure to degrade hnRNPL promoted ovarian cancer cell proliferation in vitro and tumor growth vivo, phenocopying the deficiency of FBXO16 in ovarian cancer.

## Introduction

Ovarian cancer refers to malignant tumors that grow on the ovaries, of which 90% to 95% are primary ovarian cancers, and 5–10% are metastatic tumors derived from other organs [1]. Although the incidence of ovarian cancer is lower than that of cervical cancer and endometrial cancer, the mortality rate exceeds the sum of cervical cancer and endometrial cancer. The five-year survival rate of ovarian cancer is below 40%, making it one of the deadliest gynecological cancers [2]. Its lethality is largely due to its aggressive characteristics and cannot be diagnosed early [3]. Therefore, most patients develop highly metastatic, infiltrative diseases at an advanced stage. Ovarian cancer is highly heterogeneous, and adenocarcinoma accounts for the majority of malignant tumors. The current treatment strategies include surgical resection followed by platinum and taxane-based chemotherapy, or neoadjuvant chemotherapy with interim cytoreduction followed by other chemotherapy [4–6]. Unfortunately, most patients will relapse to platinum resistance within 36 months and the molecular etiology of ovarian cancer remains elusive.

Heterogeneous ribonucleoproteins (hnRNPs) are a class of RNA-binding proteins, including approximately 20 proteins. hnRNPL is first identified as a member of the hnRNP family and an important component of the nuclear hnRNP complex [7]. hnRNPL is mainly located in the nucleus and regulates the synthesis, transport, and processing of messenger RNA (mRNA), regulating diverse biological processes [8–11]. hnRNPL is also highly expressed in a variety of tumors and plays a vital role in tumor progression [12–16]. hnRNPL promotes various biological processes of tumor cells, including proliferation, clonogenic survival, and invasion [17]. However, its post-translational regulation by ubiquitin-mediated proteolysis and the cellular machinery that is responsible for its proteasomal degradation is unknown.

Cullin-ring ligases (CRLs) comprise the largest known category of ubiquitin ligases that have an enormous impact on cellular regulation. It consists of Cullin, the Rbx/Roc Ring finger protein, and substrate recognition subunits, which can regulate the degradation of many regulatory proteins in cells [18]. Seven members of the Cullin family have been reported, including Cullin1, Cullin2, Cullin3, Cullin4A, Cullin4B, Cullin5, and Cullin7. Each Cullin member can form a CRL complex with other components [19]. F-box protein (FBP) is the substrate recognition unit of the CRL1 or SCF (SKP1/Cullin1/F-box protein) protein complex. Nearly 70 F-box proteins have been identified in humans and can be divided into three categories, including FBXW (containing a WD40-repeat domain), FBXL (containing a leucine-rich-repeat domain), and FBXO (containing another type of protein interaction domain or no recognizable domain), based on their COOH-terminal domain [20]. FBPs can recruit substrates through protein-protein interactions, thereby promoting the ubiquitination and degradation of substrates. Overexpression or mutations (deletions or point mutations) of certain FBPs have been associated with cancer progression [21]. In addition, mutations in the amino acid sequence of the substrates protein that affect FBP recognition may contribute to cancer development [22]. Therefore, the identification of FBPs with significant expression differences in ovarian cancer, as well as their ubiquitinated degradation substrates and regulated signaling pathways, can help to further understand the occurrence and development of ovarian cancer [23].

Here, we report that FBXO16 is overexpressed in ovarian cancer and has potent tumor-suppressive effect by targeting a heterogeneous nuclear ribonucleoprotein for ubiquitination dependent degradation.

## Materials and methods

### Ethics statement

Four-week-old male BALB/cA nude mice were purchased from National Rodent Laboratory Animal Resources (Shanghai, China). All mice were kept in a specific pathogen-free facility and all animal experiments were conducted in accordance with protocols approved by the Animal Care and Use Committee of the First Affiliated Hospital of Zhengzhou University. For xenografts assay, 1 × 10^7^ ovarian cancer cells were suspended in 50 µl of DMEM medium, mixed 1:1 with Matrigel and injected into the flanks of the nude mice for up to a month. Tumor sizes were measured by a caliper and calculated using the formula length × width 2 × 1/2. Tumor weights were measured after mice were sacrificed.

### Cell culture and tissue samples

HEK293T, human ovarian cancer cells A2780, OVCAR8 and SKOV3 were obtained from the American Type Culture Collection (ATCC) and tested for mycoplasma contamination. HEK293T and A2780 cells were cultured in high-glucose DMEM (Invitrogen, CA, USA) containing 10% fetal bovine serum (FBS) at 37 °C in 5% CO_2_. OVCAR8 and SKOV3 cells were grown in RPMI medium (Invitrogen, CA, USA) containing 10% FBS at 37 °C in 5% CO_2_. Sixty-eight paraffin-embedded specimens of human ovarian cancer were obtained from the First Affiliated Hospital of Zhengzhou University and with appropriate patient consent. Immunohistochemical (IHC) staining of the paraffin-embedded tumor tissues was performed using anti-FBXO16 (NBP1-57614, Novus Biologicals, U.S.A.) and anti-hnRNPL (sc-32317, Santa Cruz, U.S.A.) primary antibodies, and an ABC Elite immunoperoxidase kit according to the manufacturer’s instructions.

### Plasmids

FBXO16 and hnRNPL genes were amplified from HEK293T cells by PCR and subcloned into pcDNA3.1-HA and pbabe-FLAG vectors. The truncations of FBXO16 and hnRNPL were generated with standard PCR and subcloned into pbabe-FLAG vectors. All plasmids were completely sequenced. The Lipofectamine 2000 (Invitrogen) was used for transfection according to the manufacturer’s instructions.

### RNA interference, RNA isolation, and quantitative real-time PCR

The Lentiviral shRNAs for FBXO16 were purchased from Merck (sigma) and the target sequences were provided as follow: FBXO16-shRNA1: 5′-GCTATTGAATGACCGGGTA-3′; FBXO16-shRNA2: 5′-CAAGCTTCCAAGGGTGTTA-3′; FBXO16-shRNA3: 5′-ACTATATTCAAATGGTGA-3′; FBXO16-shRNA4: 5′-TGTAATCGCTGACGTTCAAC-3′. Total RNA from cell lysate was extracted by using TRIzol reagent (Invitrogen, USA). Oligo-dT was used for the first-strand cDNA synthesis, using a cDNA synthesis kit (Takara). The real-time PCR was performed by using SYBR Premix Ex Taq (Takara, Dalian, China). GAPDH was used as an internal control. Differences in gene expression were calculated using 2-ΔΔCt method. Primers used in this study were list as follows: FBXO16 forward, 5′-AGGATGGATTTGTAATCGCTGAC-3′; reverse, 5′-CGAAAAGCTGATAAAGGGGACT-3′. hnRNPL forward, 5′-TACGCAGCCGACAACCAAATA -3′; reverse, 5′-CTCCGGGAGTCATCCGAGT -3’. PLD1 5′-GAGCCACGGGTAAATACCTCT-3′; reverse, 5′-CCGCGTGTCCAGATTTTCTATG-3′. IGF1R 5′-TCGACATCCGCAACGACTATC-3′; reverse, 5′-CCAGGGCGTAGTTGTAGAAGAG-3′. RIN1 5′-GCACCTGGCGAGAGAAAAG-3′; reverse, 5′-TAGATTTCCGCACGAGGAACG-3′. SHC1 5′-TACTTGGTTCGGTACATGGGT-3′; reverse, 5′-CTGAGTCCGGGTGTTGAAGTC-3′. CCND1 5′-GCTGCGAAGTGGAAACCATC-3’; reverse, 5′-CCTCCTTCTGCACACATTTGAA-3’. GSK3B 5′-GGCAGCATGAAAGTTAGCAGA-3′; reverse, 5′-GGCGACCAGTTCTCCTGAATC -3′. FOSL1 5′-CAGGCGGAGACTGACAAACTG-3′; reverse, 5′-TCCTTCCGGGATTTTGCAGAT-3′.

GAPDH forward, 5′-ACAACTTTGGTATCGTGGAAGG-3′; reverse, 5′-GCCATCACGCCACAGTTTC-3′.

### CRISPR/Cas9 knock out (KO) cell lines

The FBXO16 and hnRNPL KO cells were generated by CRISPR/Cas9 technology. Primers were list: FBXO16: 5′-GTGCACACATGTGCTGGCATGGG-3′; hnRNPL: 5′-GGCGGTGGCCGCTACTACGGCGG-3′. SKOV3 cells were transfected with indicated PX459 vectors for 48 h and selected with 2 µg/ml puromycin for up to two weeks. Single clones were selected and the KO efficiency was verified by western blot assay.

### Immunoprecipitation (IP)

The IP process has been described previously [24, 25]. Briefly, cells were lysed with IP buffer (150 mM NaCl, 20 mM Tris-HCL PH8.0, 0.5 mM EDTA, and 0.5% NP-40) with protease inhibitor cocktail and phosphorylate inhibitor for 30 min on ice. Cell lysates were sonicated for 10 min and centrifuged for 30 min. For endogenous IP, the filtered supernatant was incubated with either anti-hnRNPL or IgG along with protein A/G beads overnight at 4 °C in a rotating wheel. For exogenous IP, the filtered supernatant was incubated with FLAG M2 beads (Sigma, USA) for 4 h at 4 °C in a rotating wheel. Immunoprecipitates were washed eight times with IP buffer and proteins were eluted by 3 × FLAG peptides (Sigma, USA). The final eluates were boiled at 95 °C for five minutes and subjected to Western blot analysis with indicated antibodies.

### In vivo Ubiquitination assay

Cells grown to 80% confluence in 15-cm plates. Cells were lysed in SDS lysis buffer containing 1% SDS, a protease inhibitor mixture, MG132 (10 μM), and 10 mM N-ethylmaleimide (NEM) and then diluted to final 0.1% SDS. (Cells were lysed in SDS lysis buffer containing 1% SDS and then diluted to final 0.1% SDS). The whole cell lysates were incubated with 20 μL TUBE2 agarose beads (Boston Biochem) for 2 h with rotation at 4 °C. Beads were washed eight times with IP buffer, and the bound proteins were eluted in 100 μL 2 × SDS sample buffer, boiled for 5 min and subjected to immunoblotting.

### Western blotting and antibodies

Cells were lysed with lysis buffer (100 mM Tris-HCl, pH 6.8, 100 mM DTT, 1% SDS, and 10% glycerol) and boiled for 10 mins. Proteins were then separated by 10% SDS-PAGE, and transferred to PVDF membranes. Membranes were blocked in 5% non-fat milk in PBS for 45 min before incubation with primary antibodies overnight at 4 °C. Membranes were washed with PBS for three times and incubated with secondary antibody for 1 h at room temperature. Primary antibodies were used as indicated: anti-FLAG M2 (1:10,000 dilution, F1804, Sigma), anti-FBXO16 (1:500 dilution, PA5-66195, Thermo Fisher Scientific, U.S.A.), anti-FBXO16 (1:500 dilution, NBP1-57614, Novus Biologicals, U.S.A.), anti-hnRNPL (1:1000 dilution, sc-32317, Santa Cruz, U.S.A.), anti-SKP1 (1:1000 dilution, sc-5281, Santa Cruz, U.S.A.), anti-CUL1 (1:1000 dilution, sc-17775, Santa Cruz, U.S.A.), anti-HA (1:5,000 dilution, H9658, Sigma), anti-p44/42 MAPK (Erk1/2) (1:1000 dilution, #4695, Cell Signaling Technology), anti-Phospho-p44/42 MAPK (Erk1/2) (1:1000 dilution, #4370, Cell Signaling Technology), anti-p38 (1:1,000 dilution, #8690, Cell Signaling Technology), anti-p38 (1:1000 dilution, #8632, Cell Signaling Technology), and anti-β-actin (1:5000 dilution, sc-8432, Santa cruz, U.S.A.).

### BrdU assay

1 × 10^3^ ovarian cancer cells were seeded in triplicate in 96-well plates. Cell proliferation was detected using a BrdU (bromodeoxyuridine) cell proliferation assay kit (Cell Signaling) according to the manufacturer’s instruction. The absorbance at 450 nm was read.

### Colony formation analysis

1 × 10^3^ ovarian cancer cells were seeded in a six-well plate and then cultured for up to 2 weeks. Cells were stained by 0.05% crystal violet, the numbers of colonies containing more than 50 cells were counted.

### Cell invasion assay

For invasion assay, 20 μl of Marigel (Corning) were even plated onto the upper chamber of a Transwell insert (Corning, NY, USA). 5 × 10^4^ ovarian cancer cells were suspended with 100 μl serum-free culture medium and seeded into the top chamber. The lower chamber was incubated with complete culture medium. Twenty-four hours later, the insert was fixed with 4% PFA for 30 min. Non-migrated cells on the top surface of the insert were removed and migrated cells on the lower surface of the insert were stained with 0.05% crystal violet. Images of cells on the Transwell membrane were taken with a microscope at ×200 magnification and cell numbers were counted.

### Statistical analyses

Data are presented as mean ± standard deviation (SD). Statistical analysis was performed with GraphPad Prism 6.0 software. The differences between groups were calculated using the Student’s *t*-test or one-way ANOVA using a Tukey post-hoc test. *P* < 0.05 were considered statistically significant. ***P < 0.001, **P < 0.01, *P < 0.05.

## Results

### FBXO16 is overexpressed in ovarian cancer and predicts a better prognosis

**Fig. 1:**
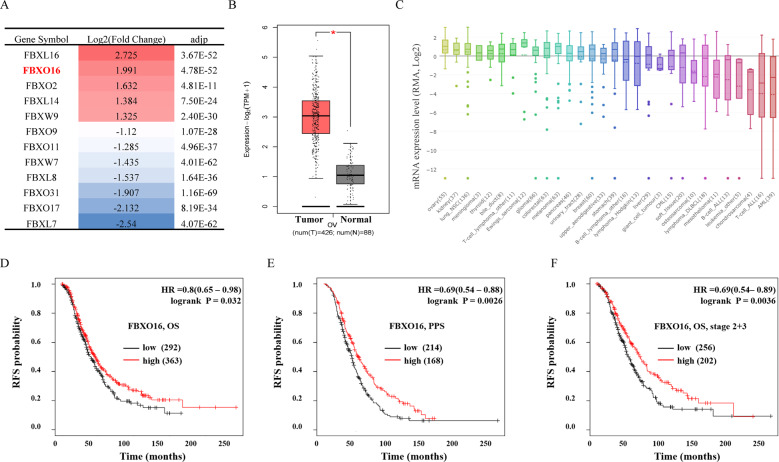
FBXO16 is overexpressed in ovarian cancer and predicts a better prognosis. **A** The differentially expressed F-box proteins (FBPs) in ovarian cancer were identified from TCGA database by employing a strict criterion (|log2FC| > 1, *p* < 0.01) base on an online analysis website GEPIA2 (http://gepia2.cancer-pku.cn/). **B** FBXO16 mRNA expression of tumor and normal tissues in TCGA ovarian cancer dataset. **C** The mRNA expression of FBXO16 was highly expressed in most ovarian cell lines in the Cancer Cell Line Encyclopedia (CCLE) database (https://portals.broadinstitute.org/ccle). **D** The overall survival (OS) curve of FBXO16 is plotted for all ovarian cancer patients in the Kaplan–Meier Plotter database (http://kmplot.com/). **E** The post-progression survival (PPS) curve of FBXO16 is plotted for all ovarian cancer patients in the Kaplan-Meier Plotter database. **F** The overall survival (OS) curve of FBXO16 is plotted for stage 2 and stage 3 ovarian cancer patients in the Kaplan–Meier Plotter database.

To investigate the role of FBPs in ovarian cancer progression, we first analyzed The Cancer Genome Atlas (TCGA) database to identify differentially expressed FBPs between 426 ovarian cancer tissues and 88 normal ovarian tissues. We employed strict criteria (|log2FC| > 1, *p* < 0.01) to screen positive candidates. compared with normal ovarian tissues, a total of 12 members of the F-box family were identified with significant abnormal expression in ovarian cancer, including 5 upregulated FBPs and 7 downregulated FBPs (Fig. 1A). In these differentially expressed FBPs, FBXL16 is the highest expressed FBP in ovarian cancer tissues, followed by FBXO16. However, it has been reported that FBXL16 cannot recruit CUL1 to form a functional SCF E3 ligase complex [26]. Therefore, we focused on FBXO16, an FBP with relatively few studies, but can form a classical SCF E3 ligase complex with CUL1 and SKP1 [27, 28]. TCGA database showed that FBXO16 mRNA expression was significantly increased in ovarian cancer tissues compared with normal ovarian tissues (Fig. 1B). We further analyzed FBXO16 mRNA expression in the Cancer Cell Line Encyclopedia (CCLE) database (https://portals.broadinstitute.org/ccle) and found that FBXO16 was relatively highly expressed in most ovarian cell lines (Fig. 1C). We further used the Kaplan–Meier Plotter database (http://kmplot.com/) to analyze the role of FBXO16 in the prognosis of ovarian cancer patients. Unexpectedly, high FBXO16 mRNA levels were significantly associated with relatively better overall survival (OS) and post-progression survival (PPS) in ovarian cancer patients (Fig. 1D, E), especially in those patients with stage 2 and stage 3 ovarian cancer (Fig. 1F), suggesting that high level of FBXO16 might have a tumor suppressor role in ovarian cancer. Together, these data indicate that FBXO16 is over-expressed in ovarian cancer, and the expression of FBXO16 can be used as a potential prognostic marker for ovarian cancer patients.

### Downregulation of FBXO16 promotes ovarian cancer cell proliferation both in vitro and in vivo

**Fig. 2:**
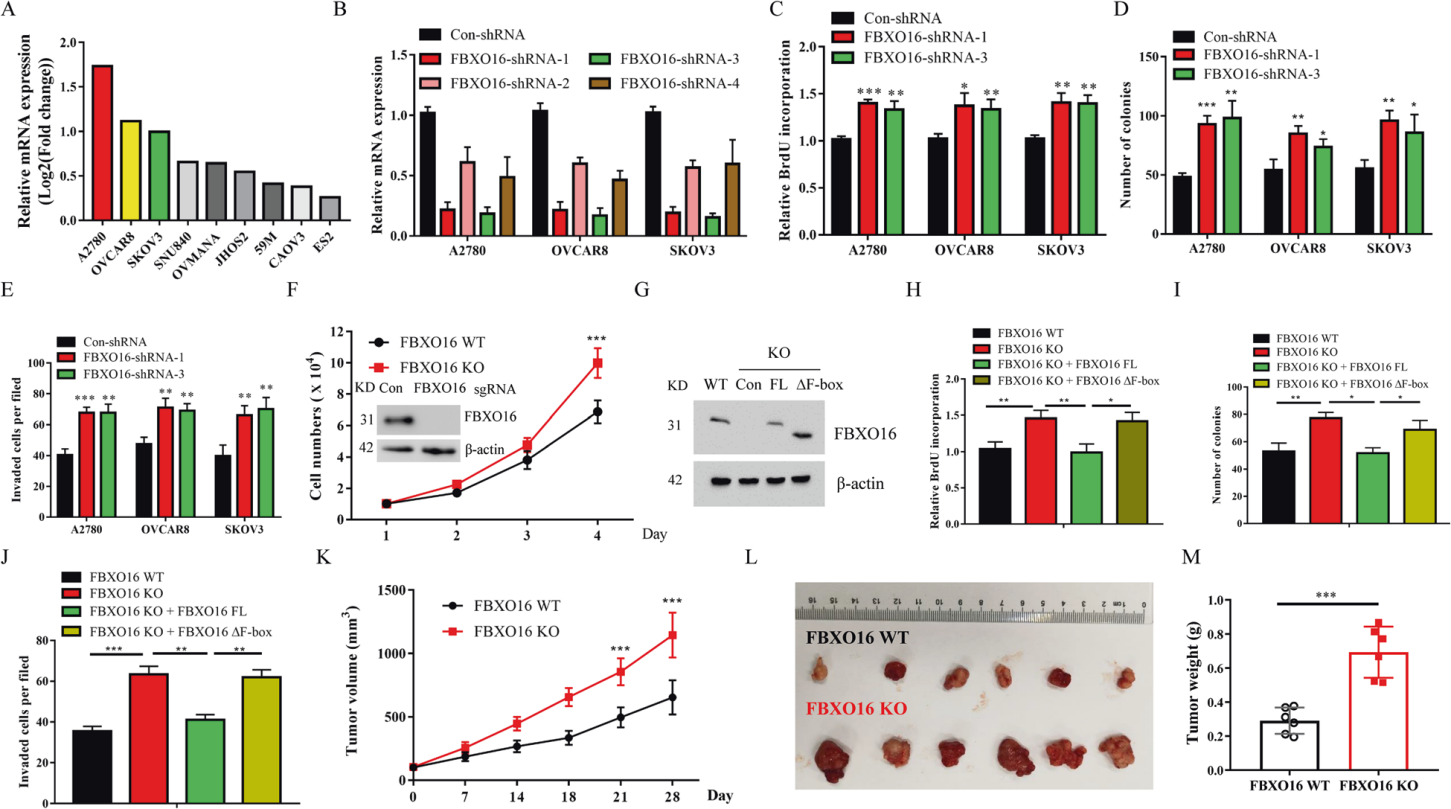
Down-regulation of FBXO16 promotes ovarian cancer cell proliferation both in vitro and in vivo. **A** The relative mRNA expression of FBXO16 in several ovarian cell lines from the Cancer Cell Line Encyclopedia (CCLE) database (https://portals.broadinstitute.org/ccle). **B** Ovarian cancer cells with or without FBXO16 silencing (FBXO16-shRNA1-4) were examined for FBXO16 mRNA expression. **C** Ovarian cancer cells with or without FBXO16 silencing were examined for BrdU cell proliferation. ***P < 0.001, **P < 0.01, *P < 0.05. **D** Cells in (**C**) were examined for colony formation. **P < 0.01,*P < 0.05. E Cells in (**C**) were examined for cell invasion. **P < 0.01. **F** FBXO16 knockout (KO) SKOV3 cells were generated by CRISPR assay and detected by western blot with indicated antibodies. The cell growth curve of control and FBXO16 KO cells was shown. ***P < 0.001. **G** FBXO16 KO SKOV3 cells infected with retrovirus coding FBXO16 wild-type (FBXO16 WT), or an F-box domain deleted mutant (FBXO16 ΔF). The cell lysates were subjected to immunoblot with indicated antibodies. **H** Cells in (**G**) were examined for BrdU cell proliferation. **P < 0.01, *P < 0.05. **I** Cells in (**G**) were examined for colony formation. **P < 0.01, *P < 0.05. **J** Cells in (**G**) were examined for cell invasion. ***P < 0.001, **P < 0.01. **K** Each nude mouse was subcutaneously injected with 1 × 10^7^ FBXO16 WT or FBXO16 KO SKOV3 cells for four weeks. Tumor growth was measured using a caliper at the indicated times after injection. n=6 for nude mice. ***P < 0.001. **L** Mice were sacrificed four weeks after transplantation. The tumors were then excised and photographed. **M** Tumor weights were measured after mice were sacrificed. ***P < 0.001.

We further determined the relative mRNA expression levels of FBXO16 in several ovarian cell lines from CCLE database and found that FBXO16 was relatively highly expressed in A2780, OVCAR8, and SKOV3 cells (Fig. 2A). To further investigate the biological function of FBXO16 in ovarian cancer, we used lentiviral-based short hairpin RNA (shRNA) to target the mRNA expression of FBXO16 in ovarian cancer cell lines. Using four independent shRNA sequences targeting different regions of FBXO16 mRNA and three ovarian cancer cell lines with relatively high FBXO16 mRNA levels (A2780, OVCAR8, and SKOV3), we were able to achieve significant down-regulation levels of FBXO16 mRNA (Fig. 2B, Supplementary Fig. 1A). qRT-PCR showed that both sh-FBXO16-1 and sh-FBXO16-3 displayed the most effective knockdown efficiency among these shRNAs we tested, inducing about 70–90% FBXO16 mRNA down-regulation in all ovarian cancer cell lines (Fig. 2B). ShRNA-mediated FBXO16 stable knockdown promoted ovarian cancer cell proliferation, clonogenic survival, and cell invasion (Fig. 2C–E, Supplementary Fig. 1B, C). All these results indicated that FBXO16 deficiency increased ovarian cancer cell malignant behavior in vitro. Consistent with these results, CRISPR-Cas9-mediated knockout (KO) of FBXO16 in SKOV3 cells promoted cell proliferation in vitro (Fig. 2F). Re-expression of wild-type FBXO16 (FBXO16 WT), but not the F-box domain deleted mutant (FBXO16 ΔF) (Fig. 2G), can impair the proliferation, clonogenic survival, and cell invasion ability caused by FBXO16 deletion (Fig. 2H–J), indicating that the tumor inhibition effect of FBXO16 depends on its E3 ligase activity. Furthermore, in vivo, malignant behavior of FBXO16 deficiency in ovarian cancer cells was determined in a xenograft mouse model. As expected, the depletion of FBXO16 dramatically enhanced the proliferation of ovarian cancer cell in vivo (Fig. 2K–M). Moreover, the immunohistochemistry (IHC) analysis for Ki67 staining, a cell proliferation marker, showed that FBXO16 deletion tumor tissues had increased Ki67 expression compared with FBXO16 WT tumor tissues (Supplementary Fig. 1D), suggesting a tumor suppressor function of FBXO16 in vivo.

### FBXO16 regulates multiple oncogenic pathways in ovarian cancer cells

**Fig. 3:**
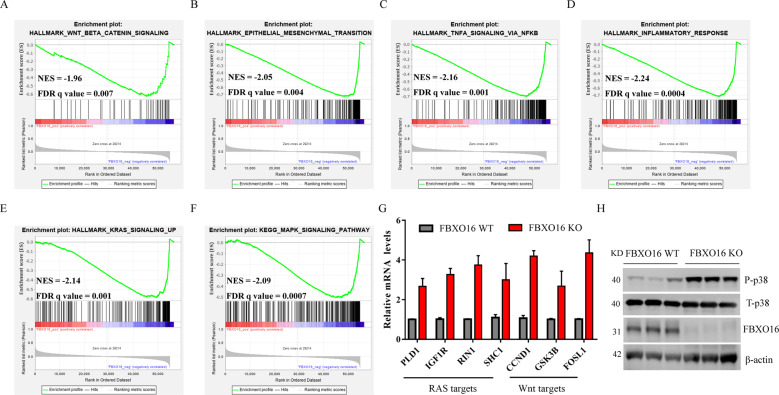
FBXO16 regulates multiple oncogenic pathways in ovarian cancer cells. **A**–**F** GSEA plot of enriched pathways in 426 ovarian cancer samples with FBXO16 expression, including HALLMARK_WNT_β-Catenin_signaling (**A**), HALLMARK_Epithelial to mesenchymal (**B**), HALLMARK_TNFA_signaling via NFKB (**C**), HALLMARK_Inflammatory response (**D**), HALLMARK_KRAS_signaling_UP (**E**), and HALLMARK_MAPK_signaling (**F**). **G** The genes associated with the RAS and WNT signaling pathways were detected by qRT-PCR in FBXO16 WT and KO SKOV3 cells. **H** The cell lysates from FBXO16 WT and KO SKOV3 cells were detected by immunoblotting with indicated antibodies. *n* = 3.

To investigate how FBXO16 affects the oncogenic property of ovarian cancer, we conducted gene set enrichment analysis (GSEA) of the RNA-seq data of 426 ovarian cancer samples from TCGA. We found that several carcinogenic pathways including WNT-β-catenin (Fig. 3A), Epithelial cell-mesenchymal transition (EMT) (Fig. 3B), TNFα (Fig. 3C), inflammatory response (Fig. 3D), RAS (Fig. 3E), and MAPK (Fig. 3F) were significantly negatively correlated with the expression of FBXO16, suggesting that high expression of FBXO16 may inhibit these carcinogenic pathways. Indeed, FBXO16 has been reported to regulate β-catenin and EMT pathways in other tumors [27, 28]. Consistent with the GSEA data, the expression of downstream genes of RAS (PLD1, IGF1R, RIN1, and SHC1) and Wnt (CCND1, GSK3B, and FOSL1) pathways were significantly increased in FBXO16 KO SKOV3 cells (Fig. 3G). Moreover, the activity of MAPK was notably increased in the absence of FBXO16 as evidenced by the increase phosphorylated p38 (Fig. 3H). Therefore, these data indicate that FBXO16 is essential to keep the inactivation of multiple carcinogenic signaling pathways in ovarian cancer cells.

### FBXO16 interacts with hnRNPL

**Fig. 4:**
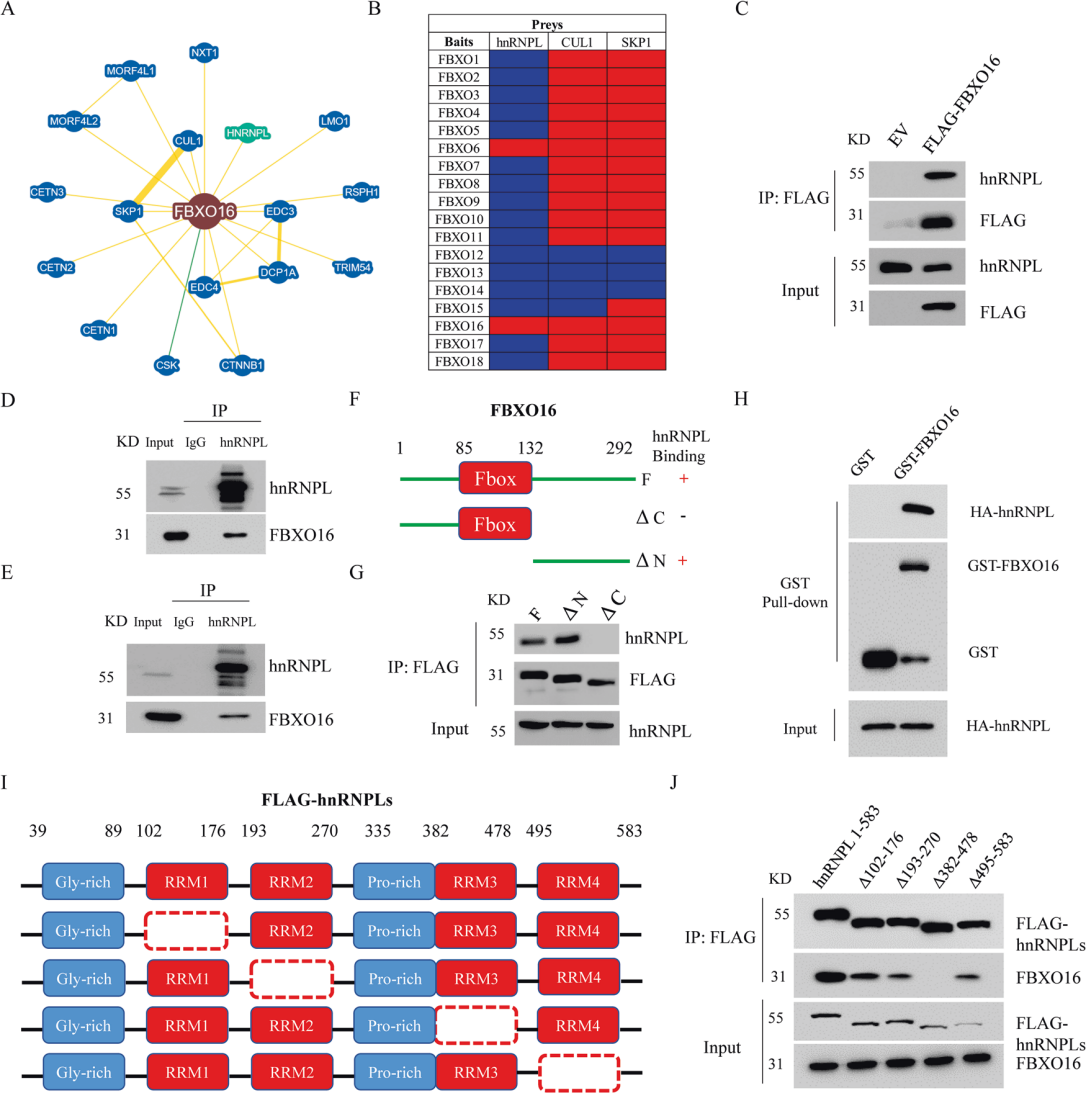
FBXO16 interacts with hnRNPL. **A** The interaction protein network of FBXO16 revealed by the BioGRID database (https://thebiogrid.org/). **B** The interaction proteins of 18 F-box proteins were collected by the BioGRID database. Red represents positive hit. Blue represents negative hit. **C** The cell lysates from HEK293T cells stable expressing empty vector (EV) or FLAG-FBXO16 were subjected to immunoprecipitation (IP) with FLAG M2 beads, followed by immunoblotting with indicated antibodies. **D** A2078 cells treated with 10 µM MG132 for 4 h were lysed and cell lysate was subjected to IP assay with anti-hnRNPL antibody, followed by immunoblotting with anti-FBXO16 antibodies. **E** IP assay was performed to detect the interaction between the endogenous FBXO16 and hnRNPL in OVCAR8 cells treated with MG132. **F** Schematic diagram of the different FLAG-tagged FBXO16 deletion mutants. **G** Cell lysates of HEK293T cells transiently expressing either full-length FLAG-FBXO16 or FLAG-FBXO16 mutants were immunoprecipitated with FLAG M2 beads, and immune complexes were blotted with anti-hnRNPL antibody. 293T cells transfected HA-hnRNPL plasmid were lysed and incubated with GST alone or GST-FBXO16 immobilized on GST-Sepharose beads. The bound proteins were then detected by immunoblotting with indicated antibodies. **H** Schematic diagram of the different FLAG-tagged hnRNPL deletion mutants. **I** Cell lysates of HEK293T cells transiently expressing either full-length FLAG-hnRNPL (1-583) or FLAG-hnRNPL mutants were immunoprecipitated with FLAG M2 beads, and the immune complexes were blotted with anti-FBXO16 antibody.

In order to investigate the molecular mechanism of FBXO16 in the regulation of ovarian cancer progress, we searched the interacting proteins of FBXO16 through the BioGRID database (https://thebiogrid.org/). A total of 17 proteins have been shown to interact with FBXO16 curated by both high and low throughput (Fig. 4A). The known SCF components CUL1 and SKP1 as well as the only reported FBXO16 substrate β-catenin are listed as FBXO16 interacting proteins. As it has been reported that FBXO16 protein is mainly distributed in the nucleus [27], we speculate that most of its substrates should also be also mainly distributed in the same place. Interestingly, we found that heterogeneous nuclear ribonucleoprotein L (hnRNPL) is listed as a FBXO16 interacting protein (Fig. 4A). hnRNPL is an RNA binding protein, mainly located in the nucleus. hnRNPL promotes various biological processes of tumor cells, including proliferation, migration and invasion by regulating various tumor-related signal pathways [17]. We have also searched the interacting proteins of 18 FBP family members in BioGRID database. The results demonstrated that although most FBPs were associated with CUL1 and SKP1, only FBXO6 and FBXO16 can bind to hnRNPL (Fig. 4B). However, our previous data showed that FBXO6 protein is mainly distributed in the cytoplasm and preferentially binds to glycoproteins, which prompted us to focus on investigating the relationship between FBXO16 and hnRNPL proteins [25, 29]. The interaction between FBXO16 and hnRNPL was confirmed by IP from FLAG-FBXO16 expressing HEK293T cell lysates (Fig. 4C). The endogenous interaction between hnRNPL and FBXO16 could also be observed in both A2780 and OVCAR8 cells (Fig. 4D–E). FBXO16 protein contains an F-box domain near its N-terminus and a C-terminus region, which is assumed to be used to recognize substrates (Fig. 4F). To map the region of FBXO16 that was responsible for interacting with hnRNPL, we generated several deletion mutants of FBXO16 protein, including the N-terminus and the C-terminus mutants (Fig. 4F). HEK293T cells transiently expressing these proteins were subjected to IP with FLAG-M2 beads. We found that the C-terminal deletion mutant of FBXO16, but not the N-terminal deletion one, failed to bind to endogenous hnRNPL (Fig. 4G), suggesting that the C-terminal region of FBXO16 is required for hnRNPL recognition. Furthermore, through GST-pull-down assay, we found that FBXO16 bound directly to hnRNPL protein (Fig. 4H). We next sought to identify the regions on hnRNPL protein that are important for FBXO16 recognition. hnRNPL contains a N-terminal glycine-rich region and four highly conserved RNA-recognition motifs (RRM) domains: RRM1, RRM2, RRM3 and RRM4 [17]. The sequence connecting RRM2 and RRM3 is abundant in proline (Fig. 4I). We then ectopically expressed hnRNPL deletion mutants in HEK293T cells and examined the ability of each mutant to bind endogenous FBXO16. We found that FBXO16 could not maintain association with hnRNPL mutant lacking the RRM3 domain (Fig. 4J). Thus, these results demonstrate that FBXO16 interacted with the RRM3 domain of hnRNPL via its C-terminal region.

### FBXO16 controls the ubiquitination and degradation of hnRNPL

**Fig. 5:**
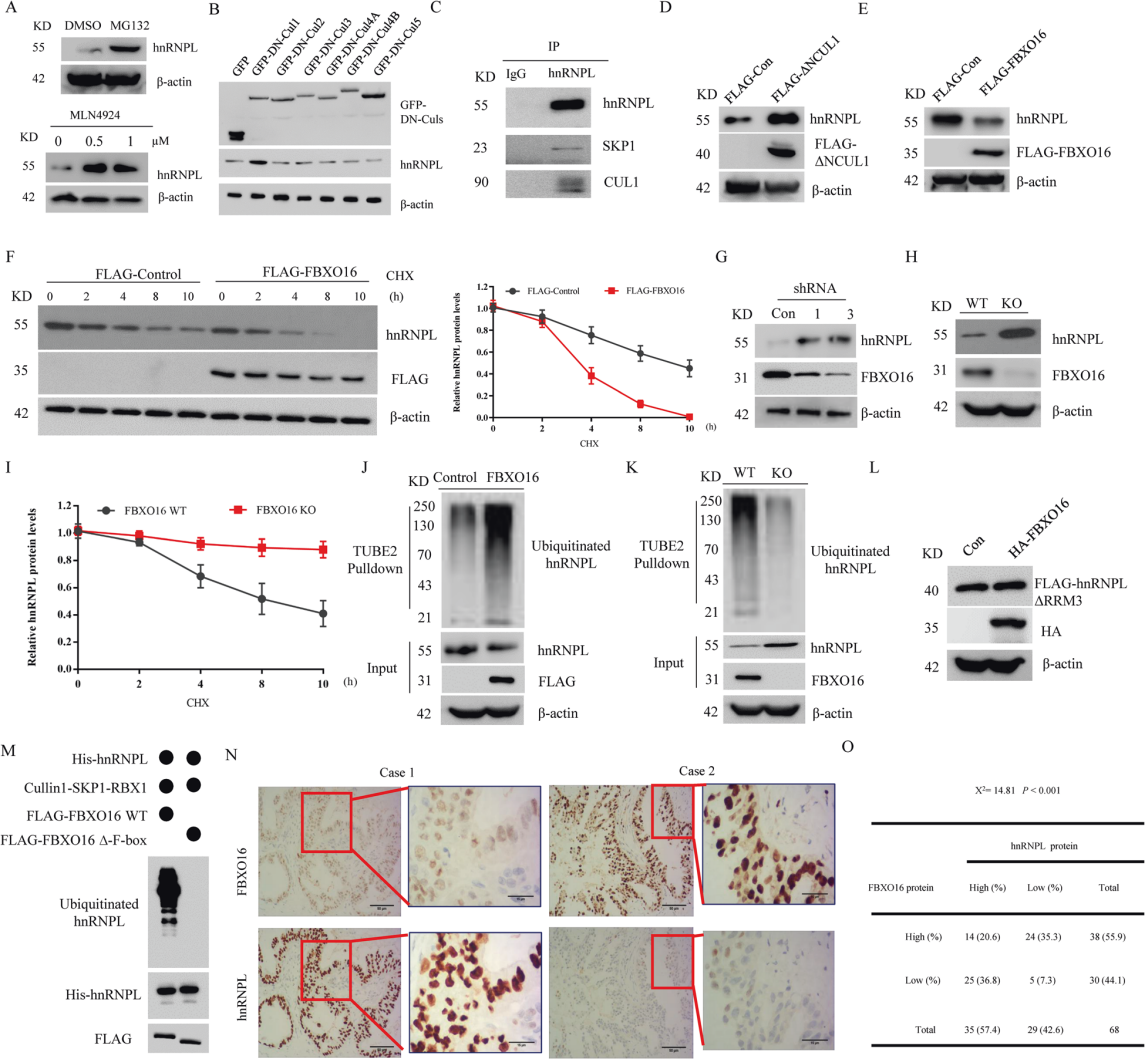
FBXO16 controls the ubiquitination and degradation of hnRNPL. **A** A2780 cells treated with 20 μM MG132 for 8 h or indicated doses of MLN4924 for 4 h were subjected to immunoblot with anti-hnRNPL antibody. **B** Immunoblotting analysis of SKOV3 cells transfected with GFP-tagged DN-Cullin1, DN-Cullin2, DN-Cullin3, DN-Cullin4A, DN-Cullin4B, DN-Cullin5, or GFP-Con vector, respectively. **C** Endogenous hnRNPL was immunoprecipitated by anti-hnRNPL antibody from A2780 cell lysate, followed by immunoblotting with indicated antibodies. **D** Cell lysates of A2780 cells transfected with control or FLAG-CUL1 (1–385) plasmids were subjected to immunoblot with anti-hnRNPL antibody. **E** Cell lysates of SKOV3 cells transfected with control or FLAG-FBXO16 plasmids were subjected to immunoblot with anti-hnRNPL antibody. **F** SKOV3 cells transfected with control or FLAG-FBXO16 plasmids were treated with 40 μM cycloheximide (CHX) for the indicated time. The whole-cell lysates (WCLs) were detected by immunoblotting with indicated antibodies. Statistic results of the western blotting analysis were obtained by image J software and normalized to actin intensities. Error bars indicate the means ± SD., *n* = 3. **G** Cell lysates of A2780 cells with or without FBXO16 silencing were subjected to immunoblot with anti-hnRNPL antibody. **H** Cell lysates of FBXO16 WT or KO SKOV3 cells were subjected to immunoblot with indicated antibodies. **I** FBXO16 WT or KO SKOV3 cells were treated with 40 μM cycloheximide (CHX) for the indicated time. The WCLs were subjected to western blot as in (**F**). Statistic results of the western blotting analysis were obtained by image J software and normalized to actin intensities. Error bars indicate the means ± SD., *n* = 3. **J** SKOV3 cells transfected with control or FLAG-FBXO16 plasmids were treated with 20 μM MG132 for 8 h. The WCLs were immunoprecipitated by Tandem Ubiquitin Binding Entity 2 (TUBE2) resin for ubiquitinated proteins enrichment and immunoblotted as indicated. **K** Control or FBXO16 KO SKOV3 cells were treated with 20 μM MG132 for 8 h. The WCLs were immunoprecipitated by Tandem Ubiquitin Binding Entity 2 (TUBE2) resin for ubiquitinated proteins enrichment and immunoblotted as indicated. **L** HEK293T cells stable expressing FLAG-hnRNPLΔRRM3 mutant were transfected with HA-FBXO16 for 36 h. The WCLs were detected by immunoblotting with indicated antibodies. **M** In vitro ubiquitination assays of recombinant His–hnRNPL protein were conducted in the presence of the Cul1-Skp1-Rbx1 complex plus FLAG-FBXO16/FLAG-FBXO16 ∆F-box proteins, E1, E2, ubiquitin, and an ATP-regeneration system were used. Samples were incubated at 30 °C for 90 min and analyzed by immunoblotting. **N** Immunohistochemical analyses of 68 specimens from ovarian cancer patients using anti-FBXO16 and anti-hnRNPL antibodies were performed. Representative images of IHC staining of tumors from two human ovarian cancer patients are presented. **O** The correlation study of FBXO16 and hnRNPL protein expression in 88 ovarian cancer samples is shown.

As a classic FBP, FBXO16 has the characteristics of regulating protein stability. We then examined the effects of FBXO16 on the stability of hnRNPL protein. We found that treatment with the proteasome inhibitor MG132 increased hnRNPL protein levels in several ovarian cancer cells (Fig. 5A, Supplementary Fig. 2A, B). Moreover, hnRNPL protein was notably accumulated in A2780 cells treated with MLN4924, a pan-CRL inhibitor [30] (Fig. 5A). To further test which Cullin is responsible for the degradation of hnRNPL, six dominant negative (DN) Cullin members were expressed into 293T cells. We showed that, among those DN-Cullin members, only DN-Cullin1 could significantly induce the accumulation of endogenous hnRNPL protein (Fig. 5B), suggesting hnRNPL is regulated by SCF E3 ligases. In addition, hnRNPL was found to interact with endogenous SKP1 and CUL1 (Fig. 5C). The expression of a dominant-negative (DN) CUL1 mutant (CUL1(1–385)) results in the accumulation of SCF substrates [31]. Indeed, the endogenous protein level of hnRNPL was also increased in the presence of DN-CUL1 (1–385) (Fig. 5D). Importantly, ectopic expression of FBXO16 in SKOV3 cells reduced endogenous hnRNPL protein expression (Fig. 5E). This reduction in protein level was due to enhanced proteolysis, as shown by the decrease in hnRNPL half-life (Fig. 5F). The protein levels of hnRNPL, but not its mRNA levels (Supplementary Fig. 2C, D), were significantly increased in FBXO16 silenced or depleted ovarian cancer cells (Fig. 5G, H), suggesting the regulation of hnRNPL by FBXO16 occurs at the post-transcriptional level. Consistent with these results, knocking out of FBXO16 significantly prolonged the half-life of hnRNPL protein in SKOV3 cells (Fig. 5I). The finding that FBXO16 reduces the protein levels of hnRNPL and that FBXO16 is able to ubiquitinate its substrates led us to study whether FBXO16 can ubiquitinate hnRNPL. We treated empty vector or FLAG-FBXO16 expressed SKOV3 cells with MG132 and then enriched ubiquitin conjugates on a tandem ubiquitin-binding entity (TUBE2) resin. Immunoblotting of the bound fraction and input (cell lysate) with antibodies against hnRNPL revealed that overexpression of FBXO16 resulted in a significant increase in ubiquitinated hnRNPL forms (Fig. 5J). In agreement, ubiquitinated hnRNPL was notably decreased in FBXO16 KO cells (Fig. 5K). Importantly, hnRNPL ΔRRM3 mutant was resistant to FBXO16-mediated ubiquitination and degradation (Supplementary Fig. 2E, Fig. 5L). The in vitro ubiquitination assay further indicated that FBXO16 WT, but not FBXO16 ΔF-box, promoted the ubiquitination of hnRNPL (Fig. 5M). Thus, these results support the hypothesis that FBXO16 promoted the ubiquitin-mediated degradation of hnRNPL in ovarian cancer cells. We next performed Immunohistochemistry (IHC) analysis to evaluate the potential association between FBXO16 and hnRNPL in 68 human ovarian cancer specimens (Fig. 5N) and a negative correlation between FBXO16 and hnRNPL proteins was observed in those ovarian cancer specimens (*χ*^2^ = 14.81, *P* < 0.001) (Fig. 5O). Together, these results suggest FBXO16 controls the ubiquitination and degradation of hnRNPL in ovarian cancer.

### FBXO16-mediated degradation of hnRNPL inhibits ovarian cancer cell proliferation

**Fig. 6:**
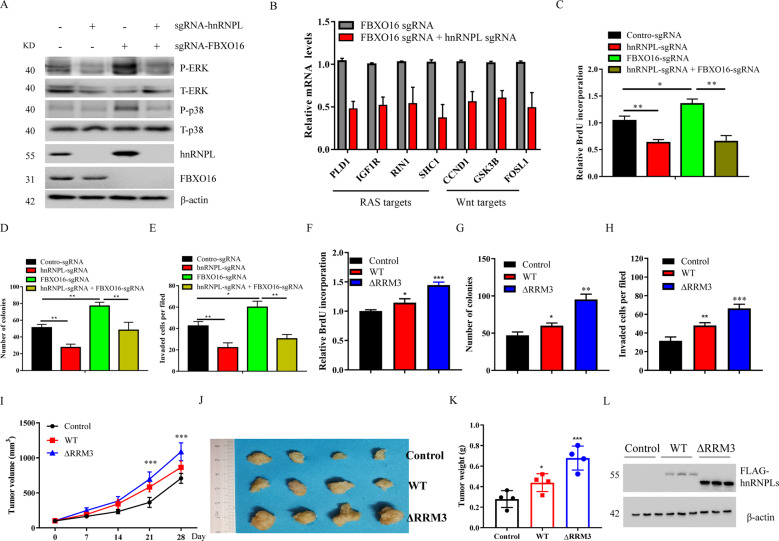
FBXO16-mediated degradation of hnRNPL inhibits ovarian cancer cell proliferation. **A** FBXO16 WT or KO SKOV3 cells were transfected with or without sgRNA against hnRNPL to knockdown hnRNPL individually or simultaneously with FBXO16. The WCLs were detected by immunoblotting with indicated antibodies. **B** The genes associated with the RAS and WNT signaling pathways were detected by qRT-PCR in FBXO16 KO SKOV3 cells with or without hnRNPL knockdown. **C** Cells in (**A**) were examined for colony formation were examined for BrdU cell proliferation. **P < 0.01, *P < 0.05. **D** Cells in (**A**) were examined for colony formation. **P < 0.01. **E** Cells in (**A**) were examined for cell invasion. *P < 0.05, **P < 0.01. *F* SKOV3 cells stable expressing control, hnRNPL WT or hnRNPL ΔRRM3 were examined for BrdU cell proliferation. *P < 0.05, ***P < 0.001. **G** SKOV3 cells stable expressing control, hnRNPL WT or hnRNPL ΔRRM3 were examined for colony formation. *P < 0.05, **P < 0.01. **H** SKOV3 cells stable expressing control, hnRNPL WT or hnRNPL ΔRRM3 were examined for cell invasion. **P < 0.01, ***P < 0.001. *I* Each nude mouse was subcutaneously injected with 1 × 10^7^ SKOV3 cells stable expressing control, hnRNPL WT or hnRNPL ΔRRM3 cells for four weeks. Tumor growth was measured using a caliper at the indicated times after injection. *n* = 5 for nude mice. ***P < 0.001. **J** Mice were sacrificed four weeks after transplantation. The tumors were then excised and photographed. **K** Tumor weights were measured after mice were sacrificed. *P < 0.05, ***P < 0.001. **L** The WCLs of tumors were subjected to immunoblot with indicated antibodies (*n* = 3).

To investigate whether hnRNPL mediates the role of FBXO16 in ovarian cancer, we further used the CRISPR/Cas9 system to knockdown hnRNPL and FBXO16 individually or simultaneously (Fig. 6A). hnRNPL knockdown notably reversed the abnormally activated MAPK, RAS, and Wnt signaling pathways in FBXO16 KO cells (Fig. 6A, B), suggesting that hnRNPL might be a critical downstream target of FBXO16 mediating the activation of these signaling pathways. Indeed, hnRNPL knockdown also largely reversed the enhanced cell proliferation, clonogenic survival, and cell invasion by FBXO16 depletion (Fig. 6C–E). Consistent with these results, stable expression of a hnRNPL ΔRRM3 mutant that cannot be recognized and degraded by FBXO16 significantly enhanced ovarian cancer cell proliferation, clonogenic survival, and cell invasion in vitro (Fig. 6F–H). Importantly, hnRNPL ΔRRM3 mutant obviously promoted tumor growth in vivo in a xenograft mouse model (Fig. 6I–L). Thus, these data suggest that FBXO16 suppresses ovarian cancer cell proliferation by promoting hnRNPL degradation.

## Discussion

In this study, FBXO16 was identified as an adaptor protein of a SCF E3 ligase complex that regulates the ubiquitination and degradation of its substrate protein hnRNPL in ovarian cancer. We found that ovarian cancer patients with relatively high expression levels of FBXO16 have a better prognosis, suggesting that FBXO16 may play a tumor suppressor role in ovarian cancer. Indeed, FBXO16 plays a critical role in the regulation of the proliferation, clonogenic survival, and cell invasion ability of ovarian cancer cells in a manner dependent on its E3 ligase activity. GSEA revealed that the expression of FBXO16 is negatively correlated with various cancer-promoting signaling pathways, suggesting that FBXO16 may regulate these signaling pathways in ovarian cancer. In line with these results, FBXO16 has been reported to regulate the WNT pathway and the EMT process through ubiquitination and degradation of β-Catenin. We also found that in FBXO16-deficient cells, the expression of the downstream genes of RAS and EMT pathways increased significantly, accompanied by notably enhanced MAPK activity. Given the critical roles of these cancer-promoting pathways in ovarian cancer, their abnormal activation reveals the biological function of FBXO16.

Since FBXO16 exerts its biological function through ubiquitination and degradation of its substrate proteins, we further searched for its downstream substrates. FBP-mediated degradation of substrate protein requires direct contact with its substrates. By analyzing the interacting proteins of FBXO16, hnRNPL was considered to be a potential substrate of FBXO16. There are several pieces of evidence to support this: (1) hnRNPL was associated with both CUL1 and SKP1 and regulated by a dominant-negative CUL1 mutant (2) FBXO16 interacted with the RRM3 domain of hnRNPL via its C-terminal region. (3) knockdown of FBXO16 delayed the turnover of hnRNPL and diminished the polyubiquitination of hnRNPL (4) hnRNPL ΔRRM3 was resistant to FBXO16-induced degradation. In addition, it has been shown that hnRNPL can regulate the inflammatory response, TNFα, and MAPK pathways [12, 32], and these are negatively correlated with FBXO16 expression. In FBXO16-deficient ovarian cancer cells, depleting hnRNPL not only inhibited cell proliferation, clonogenic survival, and cell invasion, but also repressed the activation of signaling pathways such as RAS, EMT, and MAPK, which were closely related to the molecular mechanism and biological function of FBXO16. More importantly, stable expressing a hnRNPLΔRRM3 mutant that cannot be recognized and degraded by FBXO16 phenocopied FBXO16 deficiency in ovarian cancer cells, indicating FBXO16 promotes the proliferation and invasion of ovarian cancer cells mainly via hnRNPL degradation. As FBXO16 can modulate and negatively correlate with many cellular signaling pathways in ovarian cancer cells, we hypothesize that FBXO16 could also regulate the polyubiquitination and degradation of other substrates, which requires further investigation to clarify. In addition, given the tumor suppressor role of FBXO16 in ovarian cancer, how FBXO16 mRNA is upregulated needs further investigation. In several kinds of hnRNPL overexpressing cancers, hnRNPL acts as an intron splicing factor that interacts with CA repeats in the 3′ untranslated region (UTR) of the Bcl-2 mRNA to prevent its degradation [33]. Increased hnRNPL expression might lead to decreased degradation of the Bcl-2 mRNA, which causes cancer [34]. It will be interested to investigate whether the FBXO16/hnRNPL axis also regulates the stability of Bcl-2 mRNA in ovarian cancer in the future.

In summary, our study found that FBXO16 is overexpressed in ovarian cancer tissues and cell lines, whereas high FBXO16 mRNA levels are significantly associated with relatively better prognosis. The high expression of FBXO16 can inactivate a variety of oncogenic signaling pathways and thus help to inhibit excessive tumor proliferation. FBXO16 was able to suppress the proliferation, clonogenic survival, and cell invasion of ovarian cancer cells by mediating hnRNPL degradation and failure to destruct hnRNPL caused uncontrol malignant behavior of ovarian cancer cells. Altogether, our study highlights a pivotal role of the FBXO16-hnRNPL axis in controlling the proliferation, survival, and invasion of ovarian cancer cells.

## Supplementary information

supplymentary Figure1-2
