# Supplementary material for: FBXO16-mediated hnRNPL ubiquitination and degradation plays a tumor suppressor role in ovarian cancer
Source: Cell Death Dis. 2021 Jul 31;12(8):758. doi: 10.1038/s41419-021-04040-9 (PMC8325689; doi:10.1038/s41419-021-04040-9)
Supplement: Supplementary file 1 — supplymentary Figure1-2 [file 41419_2021_4040_MOESM1_ESM.docx]

**Supplementary data**

**Figure S1 Relative to Figure 2.**

**Down-regulation of FBXO16 promotes ovarian cancer cell proliferation both in vitro and in vivo.**

**
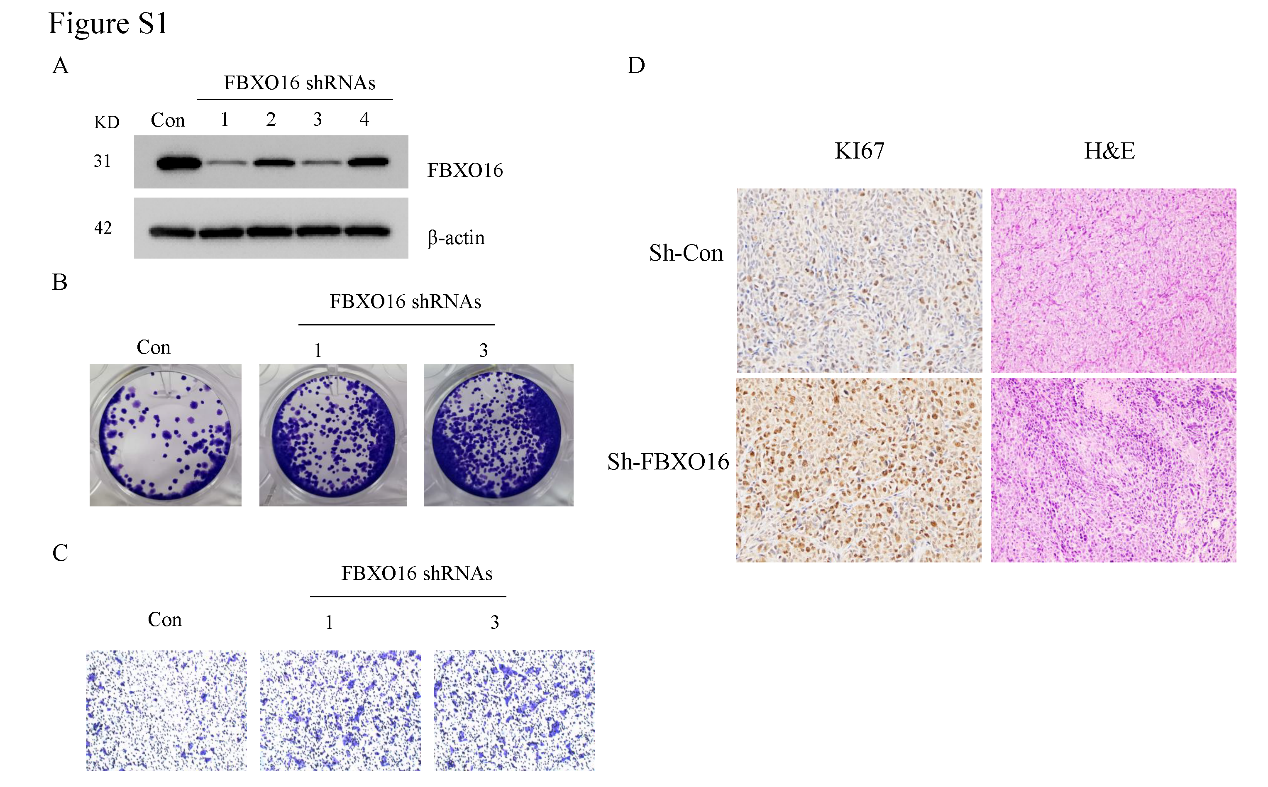
**

1. A2780 cells with or without FBXO16 silencing (FBXO16-shRNA1-4) were subjected to immunoblot with anti-FBXO16 antibody.
2. A representative colony formation picture for cells in (A).
3. A representative cell invasion picture for cells in (A).
4. The immunohistochemistry (IHC) analysis for Ki67 staining and H&E staining in both FBXO16 WT and FBXO16 deletion mice tumor tissues.

**Figure S2 Relative to Figure 5.**

**FBXO16 controls the ubiquitination and degradation of hnRNPL.**

**
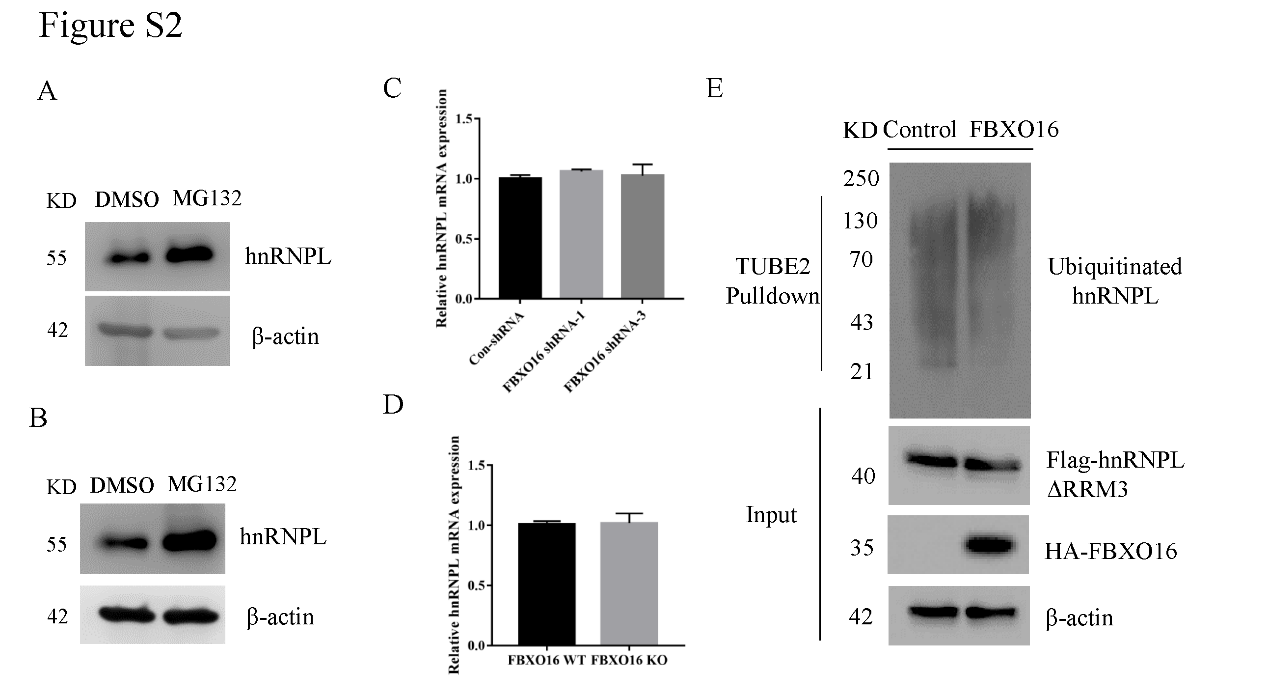
**

A. SKOV3 cells treated with 20μM MG132 for 8 hours were subjected to immunoblot with anti- hnRNPL antibody.

B. OVCAR8 cells treated with 20μM MG132 for 8 hours were subjected to immunoblot with anti- hnRNPL antibody.

C. The mRNA expression of hnRNPL in A2780 cells with or without FBXO16 silencing were detected by qRT-PCR.

D. The mRNA expression of hnRNPL in FBXO16 WT or KO SKOV3 cells were detected by qRT-PCR.

E. HEK293T cells stable expressing Flag-hnRNPLΔRRM3 mutant were transfected with HA-FBXO16 for 36 hours. The WCLs were immunoprecipitated by Tandem Ubiquitin Binding Entity 2 (TUBE2) resin for ubiquitinated proteins enrichment and immunoblotted as indicated.


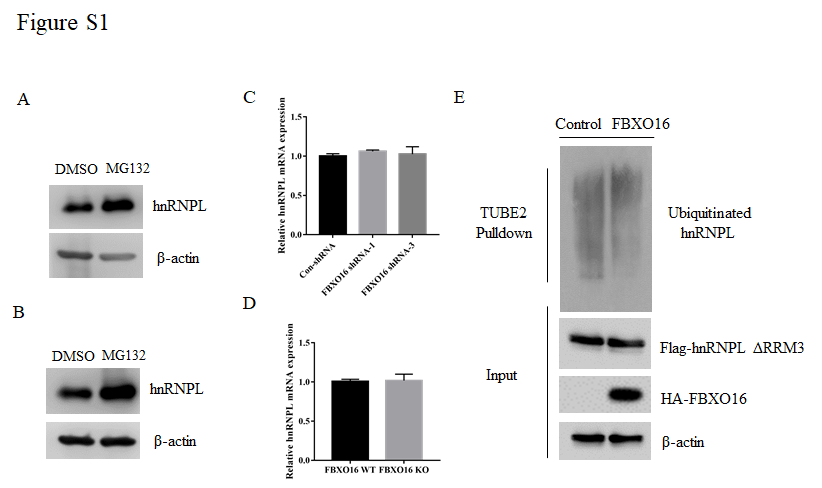


1. SKOV3 cells treated with 20μM MG132 for 8 hours were subjected to immunoblot with anti- hnRNPL antibody.
2. OVCAR8 cells treated with 20μM MG132 for 8 hours were subjected to immunoblot with anti- hnRNPL antibody.
3. The mRNA expression of hnRNPL in A2780 cells with or without FBXO16 silencing were detected by qRT-PCR.
4. The mRNA expression of hnRNPL in FBXO16 WT or KO SKOV3 cells were detected by qRT-PCR.
5. HEK293T cells stable expressing Flag-hnRNPLΔRRM3 mutant were transfected with HA-FBXO16 for 36 hours. The WCLs were immunoprecipitated by Tandem Ubiquitin Binding Entity 2 (TUBE2) resin for ubiquitinated proteins enrichment and immunoblotted as indicated.
